# Illness management and recovery (IMR) in Danish community mental health centres

**DOI:** 10.1186/1745-6215-12-195

**Published:** 2011-08-17

**Authors:** Helle Stentoft Dalum, Lisa Korsbek, John Hagel Mikkelsen, Karin Thomsen, Kristen Kistrup, Mette Olander, Jane Lindschou Hansen, Merete Nordentoft, Lene Falgaard Eplov

**Affiliations:** 1 Competence Center Rehabilitation, Recovery & Shared Care, Mental Health Centre Ballerup, Ballerup Boulevard 2, 2750 Ballerup, Denmark; 2 Community Mental Health Centre Frederiksberg-Vanløse, Mental Health Centre Frederiksberg, Nimbusparken 24, 1.sal, 2000 Frederiksberg, Denmark; 3 Community Mental Health Centre Ballerup-Egedal-Herlev, Mental Health Centre Ballerup, Denmark; 4 Mental Health Centre Frederiksberg, Ndr. Fasanvej 57-59, 2000 Frederiksberg, Denmark; 5 Copenhagen Trial Unit, Centre for Clinical Intervention Research, Department 3344, Rigshospitalet, Copenhagen University Hospital, Blegdamsvej 9, 2100 Copenhagen, Denmark; 6 Mental Health Centre Copenhagen, Bispebjerg Bakke 23, 2400 Copenhagen NV, Denmark

## Abstract

**Background:**

Schizophrenia and bipolar disorder are severe mental illnesses that can have a significant disabling impact on the lives of people. Psychosocial interventions that stress hope and recovery as a part of a multi-dimensional approach are possibly indicated to support people with severe mental illness in facilitating recovery. Illness Management and Recovery (IMR) is a curriculum-based psychosocial intervention designed as structured program with a recovery-oriented approach. The aim of IMR is to rehabilitate people with severe mental illnesses by helping them acquire knowledge and skills in managing their illness and achieve personal recovery goals. Previous randomised clinical trials indicate that IMR can be implemented with a good effect and a high fidelity though further trials are crucial to demonstrate the potential effectiveness of IMR.

**Methods/Design:**

The trial design is a randomised, assessor-blinded, multi-centre, clinical trial of the IMR program compared with treatment as usual for 200 participants diagnosed with schizophrenia or bipolar disorder under the care of two community mental health centres in the Capital Region of Denmark. The primary outcome is level of functioning at the end of treatment. The secondary outcomes are disease symptoms; use of alcohol/drugs; individual meaning of recovery; hope; hospital admissions and out-patient psychiatric treatment at the end of treatment and the abovementioned and level of functioning at follow-up 21 months after baseline.

**Discussion:**

If the results of this trial show IMR to be effective these positive results will strengthen the evidence of IMR as an effective comprehensive psychosocial intervention with a recovery-oriented approach for people with severe mental illness. This will have significant implications for the treatment and recovery of people with severe mental illness.

**Trial registration:**

Registration number NCT01361698.

## Background

Schizophrenia and bipolar disorder are severe mental illnesses that impact people's lives in many disabling aspects. Research indicates that medication alone is not sufficient to help people with these diagnoses but has to be a part of a multi-dimensional approach complemented with evidence-based psychosocial interventions in a more comprehensive rehabilitation model [1,2]. Psychosocial interventions that stress coping and personal goals may contribute to facilitating recovery from the profound functional and social deficits characterising people with schizophrenia and bipolar disorder [3-5].

A recovery-oriented approach to these severe mental illnesses holds that individuals are more than the sum of their symptoms and that recovery involves a redefinition of one's illness as only one aspect of a multi-dimensional sense of self [6].

Illness Management and Recovery (IMR) is a curriculum-based, standardized program based on a recovery-oriented approach to rehabilitation for people with severe mental illnesses. The program is designed by Kim Mueser et al. as an evidence-based practice based on the principles of recovery to help people with severe mental illnesses to set individual meaningful goals for their lives and gain illness self-management skills and thereby contribute to their individual recovery-process [7]. By collecting the evidence of different empirically supported practices including psycho-education, relapse prevention, behaviour training to improve medication adherence, coping skills training and social training, IMR was developed as a full-ranged rehabilitation program and consolidated into a single standardised program for study and dissemination.

**Figure 1:**
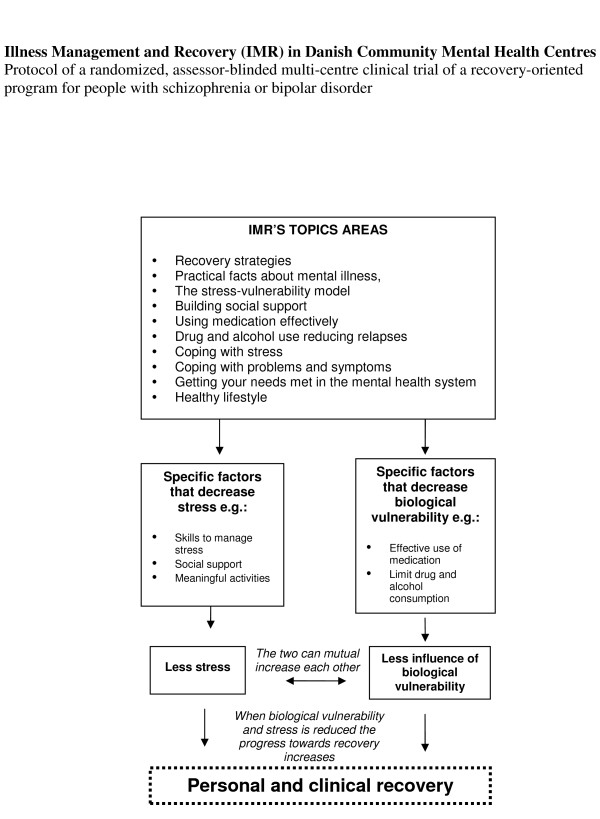
**The theoretical foundation for Illness Management and Recovery**. The trans-theoretical model and the stress-vulnerability model are the theoretical underpinning of the IMR program.

The theoretical foundation for the IMR program is the trans-theoretical model and the stress-vulnerability model. The trans-theoretical model assumes that human change developed over a series of stages and by motivating people in the stage they are in through the IMR program they can easier succeed in achieving their own personal recovery goals [8,9]. The stress-vulnerability model builds on the assumption that the course of severe mental illness is determined by an interaction of biological vulnerability, stress and coping. The aim of IMR is to interrupt the circle of stress and vulnerability that leads to poor functioning and relapse [10,11], see Figure 1.

The core values of IMR are hope, personal choice, collaboration, respect, and recognizing people as experts in their own experience of mental illness. First and foremost, the process of teaching Illness Management and Recovery involves conveying a message of hope and optimism, so that people with mental illness believe that they can reach their own goals and begin a progress of recovery. Non-controlled studies of IMR have provided some support for the effectiveness and feasibility of the program [12-14]. The effectiveness has been tested in a few randomised trials with various settings [15-18] and these trials indicate that IMR in group level can be implemented with a good effect and a high fidelity to the program curriculum [19,20]. Due to methodological limitations in the previous trials regarding the blinding process, follow-up assessments and the power of the sample size further trials are crucial to prove the effect.

In the present IMR trial, the following alternative hypotheses will be tested: Patients in the IMR program will have improved at least 6 points on the Global Assessment of Function scale (GAF-F) compared with patients receiving treatment as usual at follow-up 9 months after baseline. Furthermore, the participants in the IMR program will show a greater improvement after 9 months in relation to symptoms, drug/alcohol addiction, relapse, rehospitalisation and treatment, knowledge about their mental disease, coping strategies, recovery and hope. Moreover the difference between the intervention groups will be sustained 21 months after baseline.

## Methods/Design

The trial design is a randomised, assessor-blinded, multi-centre, clinical trial of the IMR program compared with treatment as usual in 200 participants diagnosed with schizophrenia or bipolar disorder under the care of two community mental health centres in the Capital Region of Denmark.

From January 2011 to December 2013 IMR will be tested in two community mental health centres with the participation of 200 patients. The duration of the trial will be four years. Recruitment to the trial has begun in January 2011 and is due for completion in February 2012. The intervention will start March 2011 and the follow-up assessments in November 2011. The participants will take part in the trial for the baseline interview, and for the follow-up interviews by 9 and at 21 months (one year after the intervention is ended). Moreover a naturalistic follow-up is planned in a period of ten years to evaluate any long-term effects. After the 21 months of follow-up the patients allocated to the control group will be offered IMR, if the program is shown to be effective.

### The experimental intervention

Patients randomised to the experimental intervention will be offered IMR plus 'treatment as usual'. The details of the IMR program has been described elsewhere [21], but will be briefly outlined below. The program is organised into 11 curriculum topic areas: recovery strategies, practical facts about mental illness, the stress-vulnerability model, building social support, using medication effectively, drug and alcohol use, reducing relapses, healthy lifestyle, coping with stress, coping with problems and symptoms, and getting your needs met in the mental health system. The first topic area is an explanation of the concept of recovery followed by an identification of personal recovery goals related to the individuals' own meaning of recovery. While the first 10 modules have been part of the IMR manual [22] from the start, the 11th module on healthy lifestyle is added later and has not been tested previously. We include this module in the IMR program on the recommendation of the founders of the program (personal communication with Professor Kim Mueser, Dartmouth University, USA).

IMR can be provided in an individual or group format, and generally lasts between four and ten months with a series of weekly sessions where mental health practitioners help the participants to develop personalized strategies for managing their mental illness and moving forward in their lives. Every session has the same routine, which means that the whole program is following a structured pattern. The curriculum topic areas are taught by IMR facilitators using a combination of educational, motivational, and cognitive-behavioural teaching strategies and homework assignments developed collaboratively with the participant. With the participants' consent, significant others (e.g. family, friends) are encouraged to be involved in helping participants learn self-management strategies and pursue their personal goals. In the program the participant's individual goals are often broken down into smaller steps to facilitate a continuously progress towards achieving the goals.

In this Danish trial IMR will be implemented in group format with 10 patients assigned to each group and two IMR facilitators, and the IMR program will require nine months of weekly sessions to complete. The curriculum of IMR has been translated into Danish prior to the intervention.

To ensure the fidelity of the intervention at the community mental health centres the IMR facilitators will:

■ Be experienced mental health clinicians with all together at least four days course in teaching IMR. The teaching will be given prior to the intervention, and again after six months and if need again after one year by a well-experienced IMR educator from USA (The Mental Health Center of Greater Manchester, New Hampshire).

■ Receive supervision from the well-experienced IMR educator via an Internet connection to begin with every second week and later on once a month.

■ Be involved in a network group for all IMR facilitators, formed to support the implementation of IMR in the two community mental health centres.

■ Be evaluated by a trained IMR facilitator from the other community mental health centre halfway through each IMR course using the IMR Fidelity Scale. The IMR Fidelity Scale consists of 13 items that are rated on a 5 point scale [22].

### The control group

Patients randomised to the control group will get 'treatment as usual' only. This means individual adapted interdisciplinary treatment including medication, individual support, occupational therapy, psycho-education and group therapy. Some of the staff members that also are IMR facilitators have the role of being primary care provider both for participants in the IMR intervention and in the control group. To ensure that the staff members are following the principles of treatment as usual when meeting patients in the control group, they can consult a task force consisting of a well-experienced psychiatrist only performing treatment as usual.

### Inclusion and exclusion criteria

Eligible participants will be adults (age 18+) of both sexes who are: 1) associated with one of the two participating community mental health centres; 2) diagnosed following the ICD-10 criteria of schizophrenia or bipolar disorder; 3) able to speak and understand Danish; 4) giving informed consent verbally and in writing.

Patients will be excluded if they have: 1) a guardian or a forensic psychiatric arrangement; 2) comorbidity with the ICD-10 criteria of the diagnoses of dementia or mental retardation; 3) a large-scale substance abuse - if later on the abuse is under control, inclusion in the trial will be possible; 4) a current home of supported housing - since the treatment as usual given to this group of patients is significant different from patients living independently; 5) a current involvement in a psycho-educational course - patients are eligible for participation in the trial after the psycho-educational course has ended if they meet the inclusion criteria at this point; 6) not given informed consent.

### Recruitment and randomisation

Patients will be recruited from two community mental health centres in the Capital Region of Denmark: Frederiksberg-Vanløse and Ballerup-Egedal-Herlev, respectively. Each potential participant is interviewed with the diagnostic tool Present State Examination by a psychiatrist or a trained psychologist to evaluate whether the patient meets the criteria of diagnosis. After informed consent is obtained for each eligible patient the baseline assessments will be conducted. After baseline assessments the participants will be randomly allocated to either IMR or continue with treatment as usual. To secure concealment of the allocation sequence the randomisation will be central and telephone-based through The Copenhagen Trial Unit. The allocation sequence will be computer-generated, using permuted blocks of a varying block size with equal allocation to the two arms. The allocation sequence is stratified by diagnosis and community mental health centre.

### Participant withdrawal

If the participant decides to withdraw from the trial they can 1) participate in the baseline interview and then withdrawal from the treatment but participate in the follow-up interviews; 2) participate in the baseline interview and then withdrawal from the treatment and not participate in the follow-up interviews 3) withdraw from the whole trial so that all data about them are deleted.

### Assessments

At baseline, the participants' socio-demographic information on education, employment, marital status, clinical diagnosis, suicide attempts, and earliest contract with psychiatric services will be collected.

### Primary and secondary outcome measures

The primary outcome of the trial is overall functioning measured by Global Assessment of Function (GAF-F) post-intervention - after nine months. The GAF scale [23] can be divided into two scales GAF-F and GAF-S (one that focuses on functioning and one that focuses on symptoms) [24]. In this trial the focus of the primary outcome is functioning which is why the GAF-F scale is used. Information about the participant's level of functioning will be obtained from interviews with participants at baseline and follow-up.

The secondary outcomes include level of symptoms, social functioning, personal recovery experience and hope, substance abuse, use of services and suicide attempts. This information is obtained through an interview with an assessor blind to the allocation. The participant fill in questionnaires about their personal recovery and hope, the primary care provider (not blinded to the allocation) fill in a questionnaire about substance abuse, adverse events, and suicide attempts. Information about service use is obtained trough the hospital records. The measurements are chosen according to a well-known distinction between clinical recovery and personal recovery [25,26]. Clinical recovery refers to the absence of symptoms, an improvement in functioning and prolonged remission. Personal recovery refers to living a fulfilling and hopeful life even with limitations caused by illness. In this trial we have divided the assessments of recovery in accordance with these two perspectives.

### Clinical recovery

All outcome measures to assess clinical recovery are continuous scales. The overall level of symptoms is assessed by the subscale of symptoms of the global measurement Global Assessment of Function - Symptoms (GAF-S) [24]. The Positive and Negative Syndrome Scale (PANSS) [27] assesses the level of an severity of positive and negative symptoms as well as the general psychopathology. This scale is primary developed to measure symptoms of people diagnosed with schizophrenia but can be useful to people diagnosed with bipolar disorder as well [28]. The scale is a 7-point rating instrument that consists of 30 items.

Hamilton Rating Scale for Depression scale assesses the level of symptoms of depression. In this trial we use the version of the scale that includes 6 items referred to as Ham D_6 _[29]which is covering the core symptoms of depression.

Young Mania Rating Scale (YMRS) [29,30] is an instrument developed to rate severity of manic episodes. The YMRS consists of 11 items and a severity rating is assigned to each rating.

Personal and Social Performance (PSP) [31] is a scale developed from the Social and Occupational Functional Assessment Scale (SOFAS) to assess psychiatric patients level of functioning [32]. The scale assesses four domains of social functioning: socially useful activities, personal and social relationships, self-care and disturbing and aggressive behaviour. Besides giving more detailed and varied information about the participants' level of function, the PSP scale is used to secure the reliability of the GAF score.

### Personal recovery

Participants are answering questionnaires to assess aspects of their personal recovery this includes continuous scales of recovery, illness management, hope and satisfaction with psychiatric services.

Illness Management and Recovery Scale (IMR Scale) is a scale especially developed to the IMR program [33]. It summarizes different areas in 14 items relevant to the recovery process including personal goals, knowledge about the illness, admissions, use of alcohol or drugs, functioning, symptoms, stress and coping. The IMR Scale exists in two separate versions: one for the patient and one for the primary care provider.

Mental Health Recovery Measure (MHRM) [34] is a measurement of recovery in a general level and not specific attached to the IMR program. MHRM includes different aspects related to the process of personal recovery. It is a 30-item self-report scale designed to assess the recovery process for people with severe mental illnesses e.g. schizophrenia and bipolar disorder.

The Adult State Hope Scale [35] is a self-report scale of hope and optimism. It consists of 6 items and is rated on a scale from 1 to 8. The scale was psychometrically tested in college students and in a community sample, but has also been shown to be appropriate for people with severe mental illnesses as well [36].

Clients Satisfaction Questionnaire [37] assesses the participants' satisfaction with the psychiatric services. This information is relevant from both groups point of views since satisfaction with services can explain eventual drop out and the level of effect on the other measures. The satisfaction of the clients also gives indications of how the mental health community should look like in the future according to its users.

### Blinding

Assessments regarding clinical recovery will be conducted by an assessor blind to treatment allocation. The assessor will go through a profound assessment training program. Issues concerning inter-rater reliability will have no bearing on this trial since only one person will be conducting the assessments. Due to the nature of the intervention neither participants nor staff can be blinded to allocation, but are strongly inculcated not to disclose the allocation status of the participant at the follow up assessments. An employee outside the research team will feed data into the computer in separate datasheets so that the researchers can analyse data without having access to information about the allocation.

### Monitoring for participant compliance

The use of treatment is registered for both the experimental intervention group and the control group. This is done to ensure the similarity of the treatment as usual that both groups are offered. The attendance at the IMR sessions is registered to monitor the participation compliance. All information regarding covariates, primary outcome, secondary measures, use of psychiatric service and attendance at the IMR sessions is recorded in a case record form. A view of the data collection is listed in table 1.

**Table 1:** Data collection

Source of collection	Assessments	Time of recording		
		
		Baseline	9 months (post intervention)	21 months (one year follow up)
Obtained trough Interview	Global Assessment of Functioning (GAF-F and GAF-S)	X	X	X
	
	Personal and Social Performance (PSP)	X	X	X
	
	Positive and Negative Syndrome Scale (PANSS)	X	X	X
	
	Hamilton Rating Scale for Depression (HAM-D6)	X	X	X
	
	Young Mania Rating Scale (YMRS)	X	X	X

Primary treatment provider fill out questionnaires	Alcohol and drug consumption	X	X	X
	
	Diagnose	X		
	
	First contact with psychiatric service	X		
	
	Suicide attempts	X	X	X
	
	Marital status	X		
	
	Housing condition	X		
	
	Education	X		
	
	Employment	X	X	X
	
	Use of treatment as usual		X	X
	
	IMR attendance - only for intervention group		X	X
	
	Life-threatening conditions (other than suicide)		X	X
	
	Illness Management and Recovery Scale - staff version	X	X	X

Patient fill out questionnaires	Illness Management and Recovery Scale - patient version	X	X	X
	
	Mental Health Recovery Measure	X	X	X
	
	Adult State Hope Scale	X	X	X
	
	Clients Satisfaction Questionnaire	X	X	

Hospital records	Suicide		X	X
	
	Death (all causes)		X	X
	
	Number of hospital admissions		X	X
	
	Length of hospital admissions		X	X
	
	Use of out-patient services		X	X

## Analysis

### Power and sample size

We are planning a trial of the continuous response variable, GAF-F, from independent control and experimental participants with one control per experimental participant. A previous study involving psycho-education in a Danish community mental health centre showed that response within each participant group was normally distributed with a standard deviation of 15 [38]. In the few previous studies using IMR or elements of IMR the effectiveness has been assessed by using the total GAF score showing a difference of 6-10 points [21,39]. Based on this knowledge we will conservatively estimate the true difference in the experimental and control group means to be 6 points on the GAF-F score. Using this estimation will require a total of 200 participants to reject the null hypothesis that the population means of the experimental and control groups are equal with probability (power) 0.8. The type I error probability associated with this test of the null hypothesis is 0.05. The power and sample size calculations have been made using the PS Power and Sample Size Calculations program version 3.0.34 [40].

The power of some the secondary measures has also been estimated with a total number of 200 participants. This showed that a sample size of 200 participants is sufficient to show a relevant effect size in PSP, PANSS and the IMR Scale corresponding to similar studies, see table 2. The remaining secondary measures have not been tested due to the fact that similar trials with these outcomes could not be obtained. Thus, the results of these analyses should be interpreted with caution, and the analyses should be considered as exploratory.

**Table 2:** Power calculations of PSP, PANSS and IMR Scale

Measure	The level of significance	Power	St. deviation in a similar study	Effect size in a similar study	Minimum sample size	Reference
PSP	0,05	0,8	14	7	128	*Nasrallah et al. 2008 *[40]

PANSS	0,05	0,8	11	5	154	*Fowler et al. 2009 *[41]

IMR scale	0,05	0,8	0,5	0,41	48	*Hasson-Ohayon et al. 2007*[17]

Note: PSP: Personal and Social Performance; PANSS: Positive and Negative Syndrome Scale; IMR Scale: Illness Management and Recovery Scale.

## Data analysis

The analysis will be performed by using the IBM SPSS Statistics version 19 for Windows. The data analysis will be based on the principle of intention-to-treat. The significance level for the analysis of the primary outcome and the secondary outcome will be 0.05.

Demographic variables will be listed in a table. The primary outcome measure is the continuous measurement GAF-F. The primary analysis of effectiveness will compare GAF-F at 9 months. The secondary analysis will compare effectiveness of the secondary outcome measures at 9 months and GAF-F and the secondary outcomes at 21 months. The analysis of differences between groups will be conducted using t-tests and analysis of the variances will be conducted using ANOVA. Repeated measures techniques may also be applied. Dichotomous outcome assessments will be analysed using logistic regression and paired dichotomous outcomes will be analysed using McNemar tests, or using logistic regression with random effects. Missing data is a potential source of bias and therefore the operation of multiple imputations will be used to address the issue of missing values. An analysis of the drop-out will be conducted to validate the complete case analyses.

## Ethical considerations

All participants are offered treatment as usual, i.e. treatment according to best practice and adapted to the individual needs of the patient. The trial will follow the international ethical guidelines of informed consent in clinical trials. Consent will be voluntary, informed and given both verbally and in writing. The participants will be informed about their rights to decline participation or to withdraw with no consequences to their future care or treatment. The participants will not receive a fee for their participation.

Signed consent forms will be dated, with a copy being given to the participant, and the original form kept with the research data in a locked filing cabinet. The trial has been reviewed and approved by The Ethics Committee in the Capital Region of Denmark (registration number H-1-2010-134), it is reported to the Danish Data Protection Agency and it is registered at http://www.clinicaltrials.gov (number NCT01361698).

Previous research does not indicate that IMR induces risk to the participating patients. All adverse events e.g. increase in symptoms, decrease in functioning, changes in alcohol/drug consumption and incidents of suicide, and also all beneficial events e.g. increase in level of function, hope or progress in recovery will be registered and reported. Participating in the Present State Examination, baseline and follow up interviews may cause some disturbance to the participants, but will be planned flexibly with possible breaks when needed. The results from this trial will contribute with evidence to improve treatment and rehabilitation of people diagnosed with schizophrenia and bipolar disorder. The results will also contribute to future evidence in relation to the IMR program. In the opinion of the research team the pros greatly counterbalance the cons in this trial.

## Discussion

The IMR trial is a randomised clinical trial to evaluate the effectiveness of the IMR-program for people diagnosed with schizophrenia and bipolar disorder in according to their level of functioning.

In this design of testing the IMR program an important factor is to insure the fidelity to the IMR program curriculum, so that the IMR program is the taught the same way, using the same slides and same topic areas across the participating community mental health centres. A strength of this trial is therefore that all IMR facilitators go through a specific course of education prior to the intervention, have profound supervision during the intervention and that we test the fidelity using the IMR fidelity scale throughout the course of each IMR group.

Another strength in the design of this trial is that a solid sample size calculation has been made, so we will be able to perform a data analysis with good strength according to the primary outcome, GAF-F. Besides this we have estimated the power of some the secondary measures with a total number of 200 participants. This showed that a sample size of 200 participants is sufficient to show a relevant effect size in the secondary outcome measurements PSP, PANSS and the IMR Scale which will strengthen the results in these aspects. Furthermore, the risk of selection bias related to allocation sequence generation and concealment is low, as the randomisation is performed centrally according to a computer-generated allocation sequence generation. The fact that the assessor of the primary outcome is blinded and that intention-to-treat analysis are going to be used as a statistically approach in the data analyses are also strengthening the design of this trial.

Some would argue that that there is a difference between the level of functioning of the two diagnoses, which is seen in studies comparing level of functioning for in-patients, though literature about this issue is inconsistent in their conclusions [41-43]. A recent study from Norway comparing Global Assessment of Function for the diagnoses schizophrenia and bipolar disorder in a mixed study population of in-patients and out-patients conclude that schizophrenia and bipolar disorder cannot be viewed as categorically different [44]. In the design of this trial we have carefully considered this aspect and have chosen to mix patients with two the diagnosis, since there some support of an equal level of functioning on the GAF score (it ranges from 51.79 to 53.00) [24,38,45] for this trial population and since patients included in the trial are admitted to the same treatment unit reflected same level of functioning. However, to secure that the compared groups are similar the allocation sequence is stratified by diagnosis and community mental health centre. This way potential difference in prognostics features between the two diagnoses and community mental health centres are accounted for, which increase the validity of comparison between the intervention and control group.

A limitation in the design of this trial is that some of the staff members both are IMR facilitators and at the same time maybe have patients that get treatment as usual. We have chosen not to force the patients to change their primary care provider after the result randomisation. This demands that the staff members have a stringent separation of what is the treatment as usual and what is treatment methods according to the IMR concept. To handle situation where staff might be confused of they are mixing the IMR concept with treatment as usual they can consult a task force consisting of a well-experienced psychiatrist only performing treatment as usual.

A limitation to the trial design is that only the assessor rating the factors associated to the aspects of clinical recovery is blind to allocation. When investigating a rehabilitation program like IMR it is not possible to blind the participant or the staff, so this might increase bias. The risk of bias could be seen according to the assessments related to personal recovery which is rated both by the primary care provider and by the participating patient since the awareness of the currently treatment might bias the outcome. On the other hand we think it is strength of this trial that we both get a subjective view of recovery when the person in recovery are to evaluate his/her own process and a more objective view with the clinical assessment from the primary care provider which have fulfilling knowledge of the patient though close contact.

## Competing interests

The authors declare that they have no competing interests.

## Authors' contributions

All authors participated in the conception and design of the trial. HSD has drafted the manuscript and has along with LK been involved in revising it critically for important intellectual content. All authors have read, revised critically and approved the final manuscript.
